# Kaurenoic Acid Possesses Leishmanicidal Activity by Triggering a NLRP12/IL-1*β*/cNOS/NO Pathway

**DOI:** 10.1155/2015/392918

**Published:** 2015-05-13

**Authors:** Milena Menegazzo Miranda, Carolina Panis, Suelen Santos da Silva, Juliana Aparecida Macri, Natalia Yoshie Kawakami, Thiago Hideki Hayashida, Tiago Bervelieri Madeira, Vinicius Ricardo Acquaro, Suzana Lucy Nixdorf, Luciana Pizzatti, Sérgio Ricardo Ambrósio, Rubens Cecchini, Nilton Syogo Arakawa, Waldiceu Aparecido Verri, Ivete Conchon Costa, Wander Rogério Pavanelli

**Affiliations:** ^1^Department of Pathological Sciences, Center of Biological Sciences, State University of Londrina, 86057-970 Londrina, PR, Brazil; ^2^Laboratory of Inflammatory Mediators, State University of Western Parana, 85605-010 Francisco Beltrão, PR, Brazil; ^3^Department of Chemistry, Center of Exact Sciences, State University of Londrina, 86057-970 Londrina, PR, Brazil; ^4^Department of Biochemistry, Federal University of Rio de Janeiro, 21941-909 Rio de Janeiro, RJ, Brazil; ^5^Nucleus of Research in Exact and Technological Sciences, University of Franca, 14404-600 Franca, SP, Brazil

## Abstract

*Leishmania amazonensis* (*L. amazonensis*) infection can cause severe local and diffuse injuries in humans, a condition clinically known as American cutaneous leishmaniasis (ACL). Currently, the therapeutic approach for ACL is based on Glucantime, which shows high toxicity and poor effectiveness. Therefore, ACL remains a neglected disease with limited options for treatment. Herein, the *in vitro* antiprotozoal effect and mechanisms of the diterpene kaurenoic acid [*ent*-kaur-16-en-19-oic acid] (KA) against *L. amazonensis* were investigated. KA exhibited a direct antileishmanial effect on *L. amazonensis* promastigotes. Importantly, KA also reduced the intracellular number of amastigote forms and percentage of infected peritoneal macrophages of BALB/c mice. Mechanistically, KA treatment reestablished the production of nitric oxide (NO) in a constitutive NO synthase- (cNOS-) dependent manner, subverting the NO-depleting escape mechanism of *L. amazonensis*. Furthermore, KA induced increased production of IL-1*β* and expression of the inflammasome-activating component NLRP12. These findings demonstrate the leishmanicidal capability of KA against *L. amazonensis* in macrophage culture by triggering a NLRP12/IL-1*β*/cNOS/NO mechanism.

## 1. Introduction

American cutaneous leishmaniasis (ACL) is a devastating illness caused by the protozoa* Leishmania* spp. ACL displays distinct clinical manifestations depending on both the parasite strain and the capability of the host to mount an effective immune response. Therefore, this disease may clinically appear in the host as cutaneous, mucocutaneous, or diffuse forms [1].

The treatment of ACL is based on a highly toxic chemotherapy with the antimonials sodium stibogluconate (Pentostam) and antimonate N-methyl-glucamine (Glucantime). In case of lack of response, second-line drugs such as amphotericin B or pentamidines are used [2]. However, these drugs frequently exhibit high toxicity, which has been related to its restricted use and resistance resulting in more restrictions in chemotherapy [2–5]. These considerations reveal the urgency to develop new therapeutic agents for the treatment of this disease.

Therefore, the search for more effective and less toxic chemotherapeutic agents for the treatment of ACL is increasing. There are various reported studies of synthetic compounds and natural products as potential sources of leishmanicidal activity [6, 7]. An interesting molecule towards this aim is kaurenoic acid [*ent*-kaur-16-en-19-oic acid] (KA), a diterpene obtained from various Brazilian plants [8, 9]. This molecule has been reported as showing a wide variety of biological activities such as antiprotozoal [10, 11], antimicrobial [12], antinociceptive [13], vasorelaxant, hypotensive [14, 15], and anti-inflammatory [13, 16, 17] activities. Moreover, some immunomodulatory properties of KA have been reported in these models.

The antiprotozoal activity of this diterpene involves its direct action in altering cell membrane integrity and mitochondrial membrane depolarization in promastigote and amastigote forms of* L. amazonensis* [18] and epimastigote forms of* Trypanosoma cruzi *[19], respectively. This direct activity of KA against the protozoa is not enough to reflect its overall potential as a therapeutic leishmanicidal drug, since* Leishmania* spp. parasites replicate intracellularly in macrophages and have several escape mechanisms against microbicidal molecules that are not dependent on the direct action of antileishmanial drugs [20–23].

Members of the Nod-like receptor (NLR) family of proteins have emerged as important innate immune sensors of pathogen-associated molecular patterns (PAMPS) and damage-associated molecular patterns (DAMPS) [24]. NLRs are the key components of the inflammasome that regulate the maturation of the potent inflammatory cytokine interleukin- (IL-) 1*β* [25].

Accordingly, Lima-Junior et al. found that the NLRP-3 inflammasome is engaged in the response against* L. amazonensis* restricting parasite replication. Additionally, IL-1*β* seems to be important for host resistance to infection by inducible (i) NOS-mediated production of NO, a major host defense mechanism against* Leishmania *spp. [26].

Taking into account the above-mentioned evidence, KA was evaluated for its* in vitro *effect on susceptible macrophages from BALB/c mice infected with* L. amazonensis *promastigote forms. Accordingly, we performed* in vitro *assays to investigate the direct effect of KA on parasites as well as its modulatory action on* Leishmania*-infected macrophages. We further investigated a putative mechanism of action of this compound as a modulator of proinflammatory molecules such as oxygen reactive species, NO, cytokines, and inflammasome.

## 2. Materials and Methods

### 2.1. Parasite

*L. amazonensis *(MHOM/BR/1989/166MJO) was used in promastigote forms, kept in culture medium 199 (Invitrogen-GIBCO) supplemented with 10% fetal bovine serum (Invitrogen-GIBCO), 1 M Hepes, 0.1% human urine, 0.1% L-glutamine, 10 U/mL penicillin and 10 *μ*g/mL streptomycin (Invitrogen-GIBCO), and 10% sodium bicarbonate (complete medium for promastigotes—CMP). Cell cultures were incubated at 25°C in 25 cm^2^ flasks.

### 2.2. Animals

Female BALB/c mice weighing approximately 25–30 g and aged 6–8 weeks were obtained from Fundação Osvaldo Cruz, FIOCRUZ, Curitiba, Brazil. Mice were kept under pathogen-free conditions and used according to protocols approved by the Ethics Committee of the State University of Londrina (protocol number 33064/2012.42). Every effort was made to minimize the number of animals used and their suffering.

### 2.3. Plant Material

KA used in this paper was obtained from* Sphagneticola trilobata*. The crude extract was obtained from dried roots, which were pulverized and extracted with dichloromethane and partitioned with* n*-hexane and ethyl acetate; all solvents were dried under reduced pressure. The hexane fraction was subjected to vacuum liquid chromatography (VLC) by increasing gradient polarity. The second fraction produced an amorphous compound (200 mg), which was washed with cold methanol and analyzed by high performance liquid chromatography (HPLC) methods, yielding 96% purity. The identification was performed by 1H and 13 C nuclear magnetic resonance (NMR), electron impact mass spectrometry (EIMS), and comparison with literature data [27]. The stock solution of KA was dissolved in 2% dimethyl sulfoxide (DMSO) (Invitrogen-Gibco). However, DMSO concentration did not exceed 0.2% in all experiments.

### 2.4. Viability of Promastigotes

The viability of* L. amazonensis* promastigote forms treated with KA was evaluated using the 3-(4,5-dimethylthiazol-2-yl)-2,5-diphenyltetrazolium bromide (MTT) assay as previously described [28]. Promastigote forms (10^6^/100 *μ*L) were incubated with different concentrations of KA (10, 30, 50, 70, and 90 *μ*M) or with KA solvent (0.2% DMSO) and maintained in culture for 24, 48, and 72 h at 25°C. Thereafter, 10 *μ*L of MTT (5 mg/mL) were added, followed by incubation for an additional 4 h at 24°C. The MTT formazan product was diluted with 300 *μ*L of DMSO, transferring to 96-well plates and measured in a spectrophotometer with absorbance determined at 550 nm. The results were expressed as percentage MTT reduction relative to the control group calculated as the following formula: (viable promastigotes)% = (OD of drug-treated sample/OD of untreated sample) × 100.

### 2.5. Cell Proliferation Kinetics

Promastigote forms (10^6^/mL) incubated in CMP were treated with different concentrations of KA (10, 30, 50, 70, and 90 *μ*M) or with KA solvent (0.2% DMSO) and cultured for 5 days at 25°C. Promastigotes were counted in a Neubauer chamber after 24, 48, 72, and 120 h.

### 2.6. Phagocytic Assay

Macrophages (5 × 10^5^/mL) were obtained from the peritoneal cavity by the injection of 2 mL of RPMI 1640 culture medium (Invitrogen-GIBCO) supplemented with 10% fetal bovine serum (Invitrogen-GIBCO) and cultured on 24-well plates containing 13 mm diameter glass coverslips. Cells were preincubated with 200 *μ*L of RPMI medium for 2 h for adherence and incubated with promastigote forms (5 : 1) for 2 h. KA (50, 70 or 90 *μ*M) or medium was added, followed by 24 h incubation at 37°C and 5% CO_2_. The cells were stained with Giemsa to establish the phagocytic index of infection (by percentage) and the parasites/macrophage (mean). The supernatant was utilized to measure the levels of malondialdehyde (MDA), total antioxidant capacity of plasma (TRAP), nitric oxide (NO), and cytokines.

### 2.7. Measurement of the Total Antioxidant Capacity of Samples (Trapping Antioxidant Parameter (TRAP))

Samples (50 *μ*L of supernatant with cells) obtained from the phagocytic assay were analyzed as previously described by Repetto et al. [29], by using the chemiluminescence-based method. Soluble vitamin E (Trolox) was employed as a standard antioxidant. The chemiluminescence curves were obtained using the Glomax luminometer (Promega), and the results were expressed in nM Trolox.

### 2.8. Measurement of Malondialdehyde Levels (MDA) by High Performance Chromatography

MDA levels were determined as resultant of oxidative stress occurrence, which causes lipid peroxidation and the production of this metabolite. Accordingly, we used HPLC as previously described by Victorino et al. [30], with slight modifications. The analyses were conducted with an Alliance e2695 HPLC (Waters, Milford, MA, USA) equipped with a SecurityGuard ODS-C18 (4 × 3.0 mm, Phenomenex), C18 reverse phase column (Eclipse XDB-C18; 4.6 × 250 mm, 5 *μ*m, Agilent), and a photodiode array detector (Photodiode Array Detector (PDA), 2998). Analyses were conducted using Empower 2 software (Waters, Milford, MA, USA). MDA standards were prepared using 1,1,3,3-tetraethoxypropane (TEP). Aliquots containing 250 *μ*L of cells + supernatants were deproteinized by adding 20% trichloroacetic acid and reacted with 1 mL of thiobarbituric acid. The mobile phase was 70% 10 mM KH_2_PO_4_ buffer, pH 7.0, and 40% HPLC-grade methanol. Readings were obtained at 532 nm, following an 8 min isocratic flow at the rate of 1 mL/min. The results were expressed in nM MDA.

### 2.9. Determination of Nitrite Levels as Estimate of NO Production

The determination of nitrite in supernatants collected from the phagocytic tests was used to measure the concentration of nitric oxide (NO) according to Panis et al. [31], with some modifications. Briefly, the supernatant aliquots were deproteinized by adding 50 *μ*L of 75 mM ZnSO_4_ and 70 *μ*L of NaOH and shaking and centrifuging for 5 min at 10,000 rpm and 25°C. The supernatant was recovered and diluted in glycine buffer (45 g/L, pH 9.7). Cadmium granules were rinsed with distilled sterile water and added to a 5 mM CuSO_4_ in glycine-NaOH buffer (15 g/L, pH 9.7), followed by 5 min incubation, and the copper-coated cadmium granules were used within 10 min. Activated granules were added to glycine buffer-diluted supernatant and the suspension stirred for 10 min. Aliquots of 200 *μ*L were recovered in appropriate tubes for nitrite determination, and the same volume of Griess reagent was added. After 10 min incubation at room temperature, tubes were centrifuged at 10,000 rpm for 2 min at 25°C and added to 96-well microplates in triplicate. A calibration curve was prepared using dilutions of NaNO_2_, and the absorbance was determined at 550 nm in a microplate reader.

### 2.10. Cytokine Determination

The supernatants obtained from the phagocytic assay were used to determine the levels of IL-1*β*, IL-12, TNF-*α*, IFN-*γ*, TGF-*β*, and IL-10 using eBioscience commercial kits capture enzyme-linked immune sorbent assay (ELISA) (San Diego, CA, USA). According to the manufacturer's instructions, absorbance was read at 450 nm using a spectrophotometer and the results are expressed in pg/mL based on a standard curve. The sensitivity of the test was 8 pg/mL for IL-1*β*, TNF-*α*, and TGF-*β*, 15 pg/mL for IL-12 and IFN-*γ*, and 32 pg/mL for IL-10.

### 2.11. Immunocytochemical Labeling for NLRP12 and iNOS

Immunocytochemistry of NLRP12 and inducible nitric oxide synthase (iNOS) was performed on coverslip-adherent cells (cells prepared according to the protocol described in the phagocytic assay) using the labeled streptavidin biotin method with the LSAB kit (DAKO Japan, Kyoto, Japan) without microwave treatment. The coverslips were incubated with 10% Triton X-100 for 1 h, washed 3 times with PBS, and treated for 40 min at room temperature with 10% BSA. In addition, coverslips were incubated overnight at 4°C with the primary antibody (anti-NLRP12 rabbit polyclonal antibody diluted 1 : 300 (Abcam, catalog number ab93113) and anti-iNOS rabbit monoclonal antibody diluted 1 : 200 (BD Biosciences, catalog number 610599)). After secondary antibody treatment (2 h, room temperature), horseradish peroxidase activity was visualized by treatment with H_2_O_2_ and 3,3′-diaminobenzidine (DAB) for 5 min. In the last step, the sections were weakly counterstained with Harry's hematoxylin (Merck). For each case, negative controls were performed by omitting the primary antibody. Intensity and localization of immunoreactivity against primary antibody used were examined in all coverslips using a photomicroscope (Olympus BX41, Olympus Optical Co., Ltd., Tokyo, Japan). Color photomicrographs of representative areas (×40 objective lens) were digitally acquired for image analysis. For determining a semiquantitative scoring, images were evaluated by using the color deconvolution tool from Image J software (NIH, USA). Pixels were categorized as previously described by Chatterjee et al. [32] as strong positive (3+), positive (2+), weak positive (1+), and negative (0).

### 2.12. cNOS Inhibition Assay

Peritoneal macrophages were challenged with* L. amazonensis* and treated with KA as described in the phagocytic assay method. Before the treatment with KA, the cells were incubated with 20 *μ*M NG-nitro-L-arginine methyl ester (L-NAME) for 2 h at 36°C and 5% CO_2_ [33], aiming to cause pharmacological blockage of constitutive NOS. The supernatants were utilized to measure NO levels (as previously described).

### 2.13. Statistical Analysis

Three independent experiments were performed, each with triplicate datasets. Data were expressed as mean ± standard error of the mean. Data were analyzed using the GraphPad Prism statistical software (GraphPad Software, Inc., USA, 500.288). Significant differences between the treatments were determined by one-way ANOVA, followed by Tukey's test for multiple comparisons. *P* < 0.05 was considered statistically significant.

## 3. Results

### 3.1. Kaurenoic Acid Exerts Leishmanicidal Effect against Promastigote and Amastigote Forms of* L. Amazonensis*

**Figure 1:**
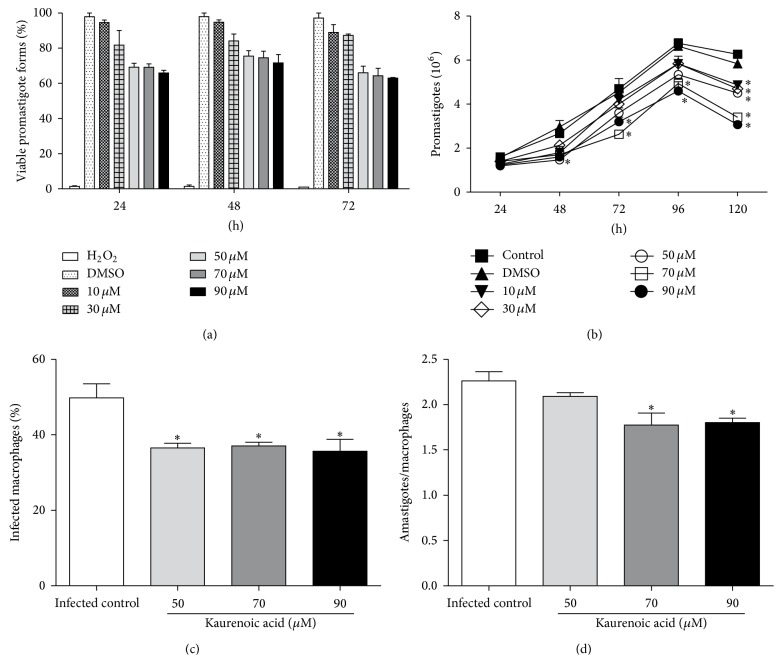
Kaurenoic acid has a leishmanicidal effect against promastigote and amastigote forms of* L. amazonensis*. MTT assay in promastigote forms of* L. amazonensis* treated with kaurenoic acid (10, 30, 50, 70, and 90 *μ*M) or 0.2% DMSO for 24, 48, and 72 h (Panel (a)). Proliferation kinetics of* L. amazonensis *promastigote forms after pretreatment for 24 h with kaurenoic acid (10, 30, 50, 70, and 90 *μ*M) or 0.2% DMSO for 24, 48, 72, and 120 h (Panel (b)). Percentage of infected macrophages (Panel (c)) and number of amastigotes per macrophage after 24 h of incubation with kaurenoic acid (50, 70, and 90 *μ*M) (Panel (d)). Data represent mean ± SEM of three independent experiments (promastigotes) and six independent experiments (amastigotes). (^∗^Significantly different from control (*P* < 0.05 compared with control group promastigotes in culture medium) (one-way ANOVA followed by Tukey's test).)

In the first set of experiments, the antileishmanial effect of KA was investigated against the promastigote forms of* L. amazonensis. *We observed that KA at concentrations of 50, 70, and 90 *μ*M reduced promastigote viability according to an MTT assay of 24 h by 30, 31, and 34%, respectively (Figure 1(a)), the reduction was maintained for 72 h. We also observed 28.2, 45.8, and 51.5% decrease in the proliferation of the promastigote forms at concentrations of 50, 70, and 90 *μ*M, respectively, after 120 h with 24 h of pretreatment (Figure 1(b)). Therefore, we chose testing all concentrations of KA for 24 h treatment of macrophages.

In attempt to verify if KA could enhance the leishmanicidal capacity of macrophages, we initially challenged these cells with promastigote forms of* L. amazonensis *for 2 h, for phagocytosis. Afterwards, the cells were treated with KA (at concentrations ranging from 50 to 90 *μ*M) for 24 h. Macrophages and amastigotes were counted to establish the phagocytic index, indicating the extent of infection as the number of parasites per macrophage.

Regarding the percentage of infected macrophages, 50, 70, and 90 *μ*M KA, respectively, caused a decrease of 26.6, 25.6, and 28.4% of infected macrophages when compared to the untreated infected macrophages (Figure 1(c)). Moreover, the mean number of amastigotes per macrophage was significantly decreased at the concentrations of 70 and 90 *μ*M, by 21.5 and 20.3%, respectively (Figure 1(d)).

### 3.2. Effect of Kaurenoic Acid on Macrophages Is Not Associated with ROS Production

In order to assess the involvement of KA in modulating the respiratory burst of macrophages during the* Leishmania* challenge, we measured the oxidative stress status of these cells by quantifying its total antioxidant capacity (TRAP) and MDA formation.

**Figure 2:**
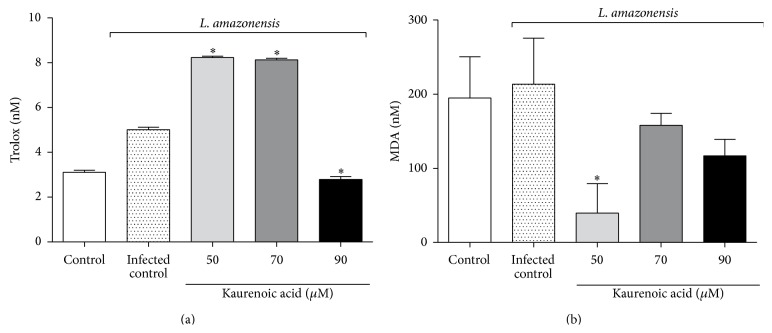
The effect of kaurenoic acid on macrophages is not associated with oxidative stress generation. Total antioxidant capacity (TRAP) (Panel (a)) and MDA levels (Panel (b)) were evaluated as markers of the oxidative status in supernatant or macrophages infected with* L. amazonensis* and treated with kaurenoic acid (50, 70, and 90 *μ*M) for 24 h. Data represent the mean ± SEM of three independent experiments. (^∗^Significantly different from infected cells (*P* < 0.05)) (one-way ANOVA followed by Tukey's test).

There was an increase in TRAP of macrophages infected with* L. amazonensis* treated with 50 and 70 *μ*M KA, while TRAP was reduced by 90 *μ*M KA compared to the infected control (Figure 2(a)). The MDA level was reduced at all KA concentrations with significant difference at 50 *μ*M KA (Figure 2(b)).

### 3.3. Kaurenoic Acid Upregulates NO Levels in a cNOS-Dependent Mechanism

**Figure 3:**
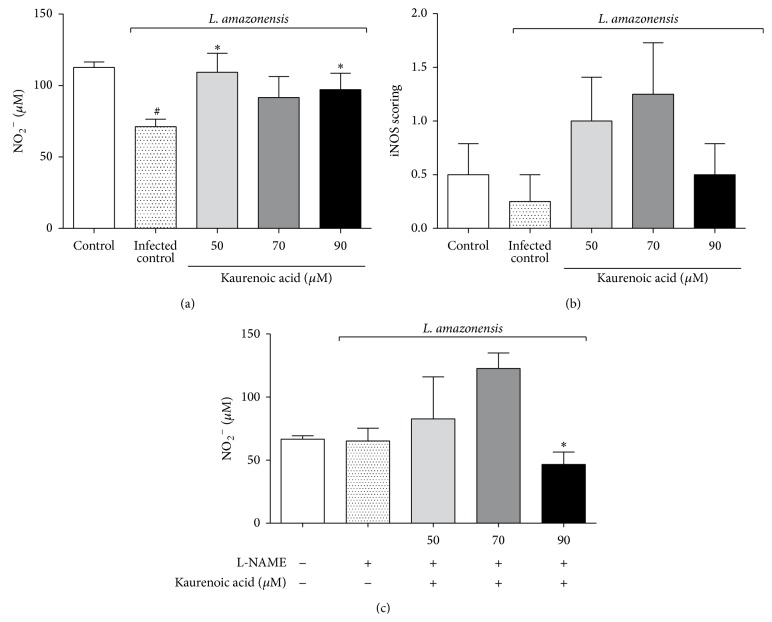
Kaurenoic acid exerted its effect by upregulating NO levels in a cNOS-dependent mechanism. NO levels (Panel (a)); immunocytochemistry scoring for inducible nitric oxide synthase (iNOS). Peritoneal macrophages infected with* L. amazonensis* and treated with kaurenoic acid (50, 70, and 90 *μ*M) for 24 h (Panel (b)). Determination of NO in peritoneal macrophages infected with* L. amazonensis* and blocked with 20 *μ*M L-NAME and treated with kaurenoic acid (50, 70, and 90 *μ*M) for 24 h (Panel (c)). Data represent the mean ± SEM of three independent experiments. (^∗^Significantly different from infected cells (*P* < 0.05). ^#^Significantly different from control cells (*P* < 0.05) (one-way ANOVA followed by Tukey's test).)

Concerning NO levels, our results showed that untreated macrophages infected with* L. amazonensis* displayed decreased levels of NO. Interestingly, the treatment with KA reestablished baseline NO levels at all concentrations tested (Figure 3(a)). Thus, we next investigated the enzymatic pathway involved in KA-induced NO production.

The expression of iNOS was assessed by immunocytochemistry. Our data showed that KA did not alter iNOS expression (Figure 3(b)).

Aiming to investigate the involvement of cNOS, we pretreated macrophages with the preferential cNOS inhibitor L-NAME. After the* Leishmania* challenge, macrophages were pretreated with L-NAME and then incubated with KA, resulting in a substantial reduction in NO production only at 90 *μ*M, indicating that the augmented NO previously observed at 90 *μ*M KA was probably dependent on cNOS activity (Figure 3(c)).

### 3.4. Kaurenoic Acid Promotes the Production of Active IL-1*β* in Macrophages Infected with* L. amazonensis*

**Figure 4:**
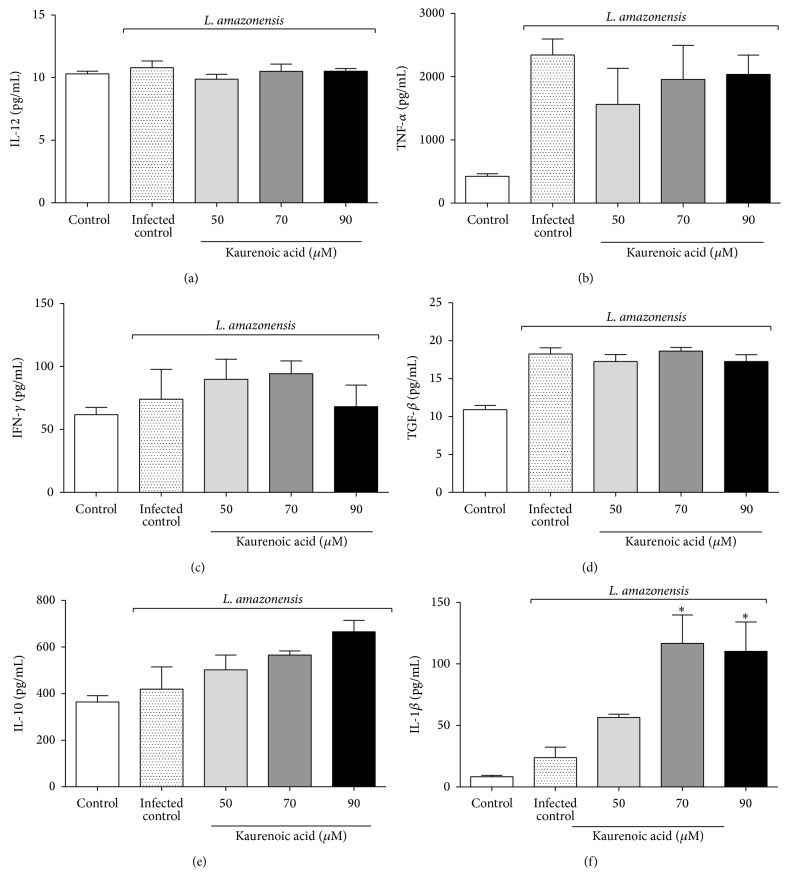
Kaurenoic acid promotes the production of active IL-1*β* in macrophages infected with* L. amazonensis*. Mapping the cytokine profiling produced* in vitro* by macrophages infected with* L. amazonensis* and treated with kaurenoic acid (50, 70, and 90 *μ*M) for 24 h determined by ELISA. IL-12 production (Panel (a)), TNF-*α* production (Panel (b)), IFN-*γ* production (Panel (c)), TGF-*β* production (Panel (d)), IL-10 production (Panel (e)), and IL-1*β* production (Panel (f)). Data represent the mean ± SEM of three independent experiments. (^∗^Significantly different from infected cells (*P* < 0.05) (one-way ANOVA followed by Tukey's test).)

In order to determine the immunomodulatory action of KA on cytokines in infected macrophages, we measured the levels of IL-12, TNF-*α*, IFN-*γ*, TGF-*β*, IL-10, and IL-1*β*. We observed that the production of IL-12, TNF-*α*, IFN-*γ*, TGF-*β*, and IL-10 was not significantly different between the KA-treated groups and the control group (Figure 4). On the other hand, 70 and 90 *μ*M KA augmented the levels of IL-1*β* (Figure 4(f)).

### 3.5. Kaurenoic Acid Upregulates NLRP12 Expression in Macrophages Infected with* L. amazonensis*

**Figure 5:**
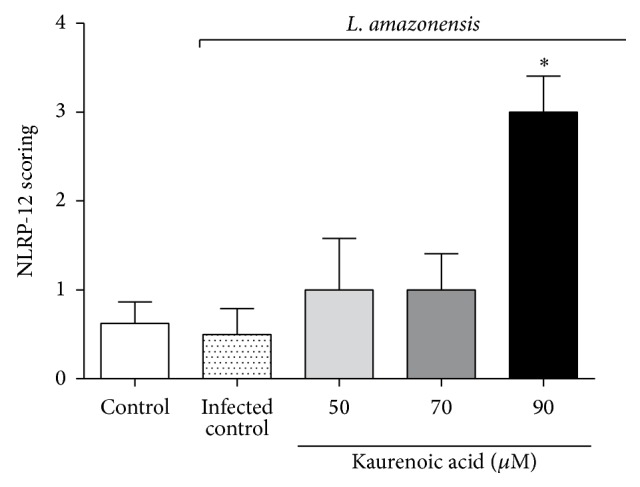
Kaurenoic acid upregulated the NLRP12 expression in macrophages infected with* L. amazonensis*. Immunocytochemistry scoring for NLRP12 in macrophages infected with* L. amazonensis* and treated with kaurenoic acid (50, 70, and 90 *μ*M) for 24 h. Data represent the mean ± SEM of three independent experiments. (^∗^Significantly different from infected cells (*P* < 0.05). (One-way ANOVA followed by Tukey's test).)

In this present study, the activation and participation of the inflammasome during the immune response to infection by intracellular pathogen were investigated. The augmented levels of IL-1*β* induced during KA treatment of infected macrophages, combined with the lack of information about this complex in ACL, led us to investigate the role of NLRP12, one member of the subfamily of NLRP innate receptors. As shown in Figure 5, KA at 90 *μ*M was able to upregulate NLRP12 expression in macrophages infected with* L. amazonensis*. These results indicate that the stimulation of macrophages with KA triggered the overexpression of NLRP12, with consequent activation of IL-1*β*.

## 4. Discussion

The success of chemotherapy in ACL is mainly dependent on two factors: the microbicidal activity of the drug and the protective immune response triggered in the host during the treatment.

In the present study, we evaluated the therapeutic potential of KA treatment, which directly inhibited the viability and proliferation of* L. amazonensis *promastigote forms (Figure 1(a)). Previous* in vitro* studies demonstrated that KA has direct antileishmanial activity against promastigote and amastigote forms of* L. amazonensis *[19, 34, 35], and the main proposed mechanism was related to mitochondrial membrane depolarization in the protozoan. However, the immunomodulatory activity of KA in the macrophage experimental leishmaniasis model still remained to be determined.

Our results indicated that infected macrophages treated with KA were more effective during the leishmanicidal response against the intracellular forms of* L. amazonensis *(Figures 1(b) and 1(c)). The solvent (0.2% DMSO) did not affect the viability and proliferation of* L. amazonensis* promastigotes (Figures 1(a) and 1(b)). These findings suggested that this diterpene was able to reverse the downregulation of the killer machinery of macrophages caused by* Leishmania* infection [20, 36].

In order to elucidate the microbicidal effects of KA, we investigated the main leishmanicidal molecules produced by macrophages. The results showed that KA treatment was not able to enhance the oxidative burst of infected macrophages. On the other hand, even though* L. amazonensis* is capable of depleting NO levels [20], the results showed that treatment with KA was able to restore the levels of this microbicidal molecule (Figure 3(a)), but no alteration in the expression of iNOS was found.

In fact, NO is the main antileishmanial molecule produced in the early macrophage response against intracellular parasites. Besides iNOS, cNOS is also an important route for NO production [37, 38]. Some studies have demonstrated that KA induces cNOS-dependent activity in the disease context [13, 14], but the involvement of a cNOS mechanism for KA in parasitic infections has not been well elucidated.

Studies have demonstrated the importance of innate immune response-triggered cytokine production during the early stages of experimental leishmaniasis and have shown that some cytokines may drive the clinical manifestation of ACL by modulating resistance or susceptibility to infection [39]. IL-1*β*, IL-12, TNF-*α*, and IFN-*γ* are essential cytokines for the development of an effective immune response against* Leishmania* spp., leading to the activation of macrophages and promoting the microbicidal effects against this parasite [23, 26, 40, 41].

In the present experimental conditions, KA was able to increase the production of IL-1*β* despite of showing no effect on other cytokines evaluated, suggesting a selective effect of KA on IL-1*β* production/maturation system. IL-1*β* is a proinflammatory cytokine that becomes active after its cleavage by the inflammasome complex [42], and when active, this cytokine helps the activation of macrophages by enhancing the response against pathogens. In fact, a recent study showed that IL-1*β* was associated with resistance to* L. amazonensis, L. braziliensis, *and* L. major* infections. IL-1*β* maturation is dependent on the inflammasome NLRP3/ASC/caspase-1 complex [26]. In addition to NLRP3, which is endogenously expressed during* Leishmania* infection, NLRP12 is an important NLR involved in the inflammatory response against parasites such as* Trypanosoma cruzi* [43]. In this sense, the present study also addressed whether the expression of NLRP12 increased during* Leishmania* infection of macrophages to determine if KA induces IL-1*β* production/maturation by a previously unrecognized mechanism.

Our findings showed that there was no induction of NLRP12 in macrophages during* Leishmania* infection. On the other hand, KA at 90 *μ*M upregulated the expression of NLRP12 in infected macrophages, thus explaining the increased production of IL-1*β* induced by KA. These data are also in line with the increased cNOS-dependent production of NO, since it has been shown that during* Leishmania* infection a NLRP3/ASC/caspase-1/IL-1*β*/cNOS/NO pathway is triggered to kill this parasite. In the present study, the data suggest that KA triggers a NLRP12/IL-1*β*/cNOS/NO killing mechanism during* Leishmania *infection of macrophages from BALB/c mice. Importantly, KA-induced the expression of a NLR (NLRP12) that is not endogenously activated to kill* Leishmania*, thereby upregulating unused endogenous mechanisms valuable to protect the host against* Leishmania* infection with additive effects for other NLRs such as NLRP3, as observed in* T. cruzi* infection [43]. Therefore, KA seems to unequivocally provide additional protective mechanisms against* Leishmania* infection. It is also possible that KA triggers similar mechanisms in other parasitic diseases, which remains to be determined.

In conclusion, the present study demonstrated that kaurenoic acid has therapeutic potential as a pharmacological approach against* Leishmania* infection. The mechanism of action of kaurenoic acid depends, at least in part, on triggering the NLRP12/IL-1*β*/cNOS/NO leishmanicidal pathway. Therefore, KA merits further preclinical and clinical studies as a possible therapy for* Leishmania* infection.
